# Local allergic rhinitis: natural history

**DOI:** 10.1186/2045-7022-3-S2-O1

**Published:** 2013-07-16

**Authors:** Carmen Rondon, Paloma Campo, Immaculada Doña, Federico De La Roca, Natalia Blanca Lopez, Miguel Blanca

**Affiliations:** 1 Carlos Haya Hospital, Malaga, Spain; 2 Carlos Haya Hospital, Allergy Service, Malaga, Spain; 3 Ciudad Real General Hospital, Allergy Service, Malaga, Spain; 4 Infanta Leonor Hospital, Allergy Service, Madrid, Spain

## Background

Local allergic rhinitis (LAR) is a common respiratory disease with a prevalence of 25.7% in rhinitis population. However, whether LAR is a first step in the development of classical allergic rhinitis (AR) with systemic atopy or not, needs to be explored. The objectives of this study were to evaluate the natural history of LAR and the new incidence of systemic atopy.

## Methods

A prospective 10-year-follow-up study has been designed to evaluate 194 LAR patients and 97 healthy controls. All LAR patients had positive response to nasal allergen provocation test (NAPT) with at least one aeroallergen. Demographic and clinical questionnaire, spirometry, skin prick testing, and serum specific IgE antibodies to common aeroallergens were evaluated yearly, and NAPT were performed at initial evaluation and after 5 and 10 years of evolution.

## Results

These data represent the results of the first 5 years of the follow-up. The majority of LAR patients were non-smoker women with moderate/severe persistent perennial rhinitis, without family history of atopy and city dwelling. At initial evaluation conjunctivitis (52.3%) and asthma (18.8%) were the most frequent comorbidities, and D. pteronyssinus (51.1%) the main specific aeroallergen detected by NAPT. After 5 years a worsening of rhinitis was detected in 26.2% patients, with increase in persistence and severity of nasal symptoms. New associations to conjunctivitis (7.9%) and asthma (5.6%) were also detected. Systemic atopy was detected by SPT and/or serum specific IgE in LAR (12/176, 6.81%) and control group (4/88, 4.5%), without significant differences.

## Conclusions

The results of the first 5-year-follow-up study show that a similar proportion of LAR patients and healthy controls developed systemic atopy, suggesting LAR and classical AR can be two independent entities. However, in order to ensure these findings, it is necessary to wait for the conclusion of the 10-year-follow-up study actually in progress.

